# Neonatal cardiogenic shock revealing obstructive cardiac Hibernoma: case report

**DOI:** 10.1186/s13019-021-01582-z

**Published:** 2021-08-04

**Authors:** Rym Gribaa, Marwen Kacem, Sami Ouannes, Wiem Majdoub, Houssem Thabet, Imen Ben Ali, Aymen Elheraiche, Mehdi Slim, Sihem Hmissa, Elyes Neffati, Taieb Cherif, Chokri Kortas, Jamli Marah, Sofiene Jerbi

**Affiliations:** 1 Cardiology Department, Sahloul University Hospital, Sousse, Tunisia; 2 Anatomic and cytopathologic laboratory, Sahloul University Hospital, Sousse, Tunisia; 3 Cardiovascular surgery department, Sahloul University Hospital, Sousse, Tunisia

**Keywords:** Cardiac tumors, Pediatric cardiology, Cardiac surgery, Hibernomas, case report

## Abstract

**Background:**

Cardiac Hibernomas are very rare benign tumors and usually remain asymptomatic. Neonatal cardiogenic shock due to cardiac tumors is extremely very rare. Until this date a few cases of cardiac hibernoma have been reported in the literature.

Transthoracic echocardiography help in the differential diagnosis, but the definitive diagnosis is histological. The management strategy is not clearly codified.

The Aim is to report and discuss the clinical features of a cardiac Hibernoma and review the relevant literature.

**Case presentation:**

We describe a case of a 2-day-old Caucasian full-term male neonate admitted in neonate intensive care with cardiogenic shock, having fluid resuscitation and inotropic drugs. Ventilatory support was started immediately with the subsequent reestablishment of normal blood pressure. Then he was transferred to the echocardiography laboratory.

Transthoracic echocardiography showed two echogenic masses in the right atrium and right ventricle.

The masses were extended to the pulmonary trunk. Pulmonary artery flow measurements showed the presence of pulmonary and tricuspid obstruction.

Surgery was rapidly considered since the baby was hemodynamically unstable.

Intraoperative evaluation showed a mass embedded in the interventricular septum that occupy the right ventricular cavity and the right atrium. The tumor involved also the chordae of the tricuspid. Partial resection was done. Tricuspid valve repair was performed by construction of new chordae from the autologous pericardium. The specimen was sent for histopathological analysis. The baby died immediately after surgery.

Histological examination of the surgical specimen revealed clear multivacuolated cells filled with lipid droplets and granular intense eosinophilic cytoplasm which confirms the diagnosis of Hibernoma.

**Conclusion:**

Cardiac Hibernomas are rare benign tumors. The prognosis and treatment strategy is closely dependent on the location, initial clinical presentation and possible complications. The prognosis can be unfavorable if the tumor was obstructive and infiltrate the myocardium.

## Background

Cardiogenic shock is a primary cardiac disorder characterized by a low cardiac output state of circulatory failure that results in end-organ hypoperfusion and tissue hypoxia. The most common etiologies include congenital heart diseases with reduced cardiac output and systemic hypotension, cardiac muscle disorders, dysrhythmias and metabolic conditions [1]. Cardiogenic shock in neonates is a rare medical emergency. Transthoracic echocardiography plays a pivotal role in the diagnosis and management of infants and children presenting with cardiogenic shock.

Hibernoma is a rare benign tumor originating from remnants of fetal brown adipose tissue.

It can be found in numerous locations in the body, especially in the thigh, shoulder, and upper back [2]. Intracardiac location have been rarely described.

Cardiac Hibernomas usually remain asymptomatic, but they can cause arrhythmia, embolisation, pericardial effusion, or blood flow obstruction.

We report a case of a 2-day-old Caucasian full-term male neonate who was admitted in neonate intensive care with cardiogenic shock due to an obstructive a cardiac Hibernoma.

## Case report

A 2-day-old Caucasian full-term male neonate was admitted in neonate intensive care with shock. There were no familial antecedents, no consanguinity and no infection or inflammation during pregnancy. However the tumor was suspected in fetal echocardiography.

At observation, he presented poor general status with marked hypotonia, immeasurable blood pressure, tachypnoea and retractions. Percutaneous oxygen saturation was 85% on room air. He had a mottled blue and white skin with prolonged capillary refill time (above 3 s). Cardiac auscultation showed rhythmic heart sounds without murmurs. On pulmonary auscultation, he had a symmetric entrance of air and without rales.

He was intubated and ventilated with conventional mechanical ventilation. We started intravenous fluid resuscitation, inotropic drugs (dobutamine), with the subsequent reestablishment of normal blood pressure. He was transferred to the echocardiography laboratory.

**Fig. 1:**
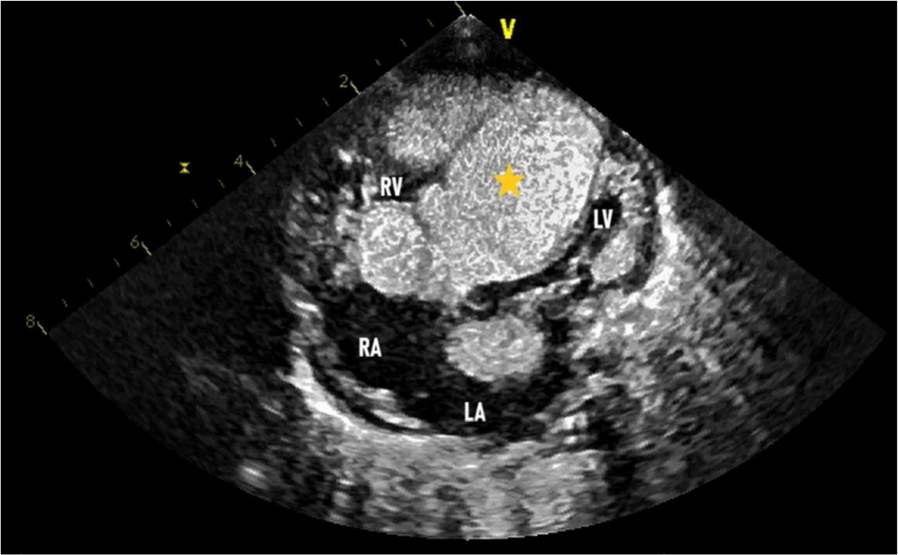
Apical Five Chamber View. Left Atrium (LA). Left ventricle (LV). Right Ventricle (RV). Right Atrium (RA). Asterisk showing the Hibernoma exerting mass effect on the LV

**Fig. 2:**
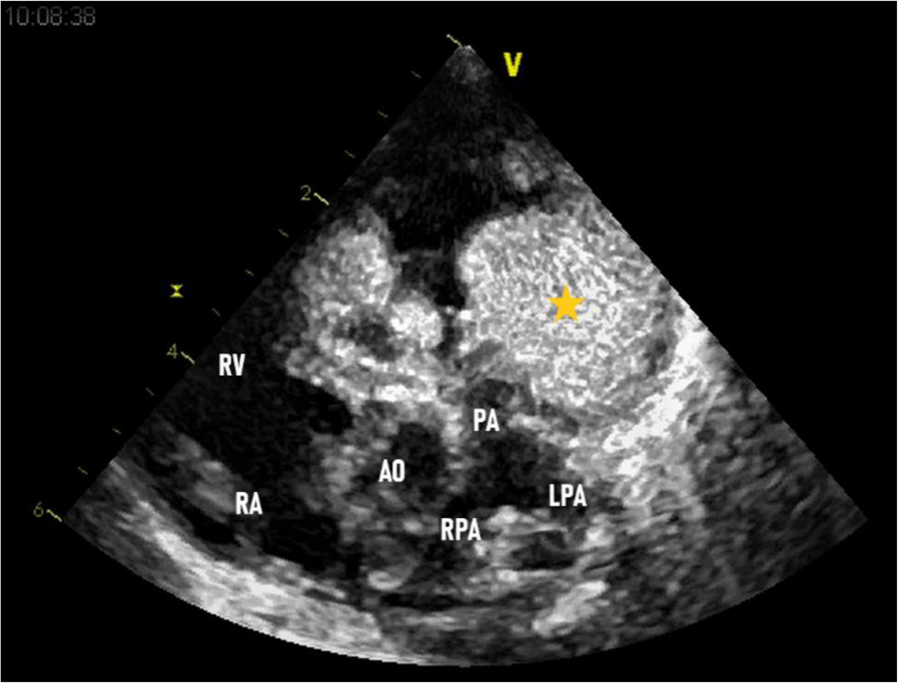
Parasternal short axis view at the level of the aortic valve. Asterisk showing the obstructive mass in the RVOT. pulmonary artery (PA), right pulmonary artery (RPA) and left pulmonary artery (LPA), right ventricle outflow tract (RVOT)

Transthoracic echocardiography showed two echogenic masses. The first one, was large, poly-lobed, and mobile mass in the right ventricle, which measured 37 mm × 29 mm. The mass was embedded in the interventricular septum (IVS) and right ventricle (RV) wall (Fig. 1). This mass was extended to the pulmonary trunk (Fig. 2). Pulmonary artery flow measurements showed the presence of pulmonary and tricuspid obstruction. The peak pressure gradient between the right ventricle and the pulmonary trunk was 60 mmHg.

The second one, was a large mass measuring 28 mm × 22 mm in the right atrium. Color Doppler flow imaging did not detect any flow signal inside the mass.

To further characterize the mass, cardiovascular Magnetic resonance imaging was indicated, however it was not performed because of unavailability of this exam in emergency. Surgery was rapidly considered since the baby was hemodynamically unstable. He was transferred operating room for emergency surgery the next day.

**Fig. 3:**
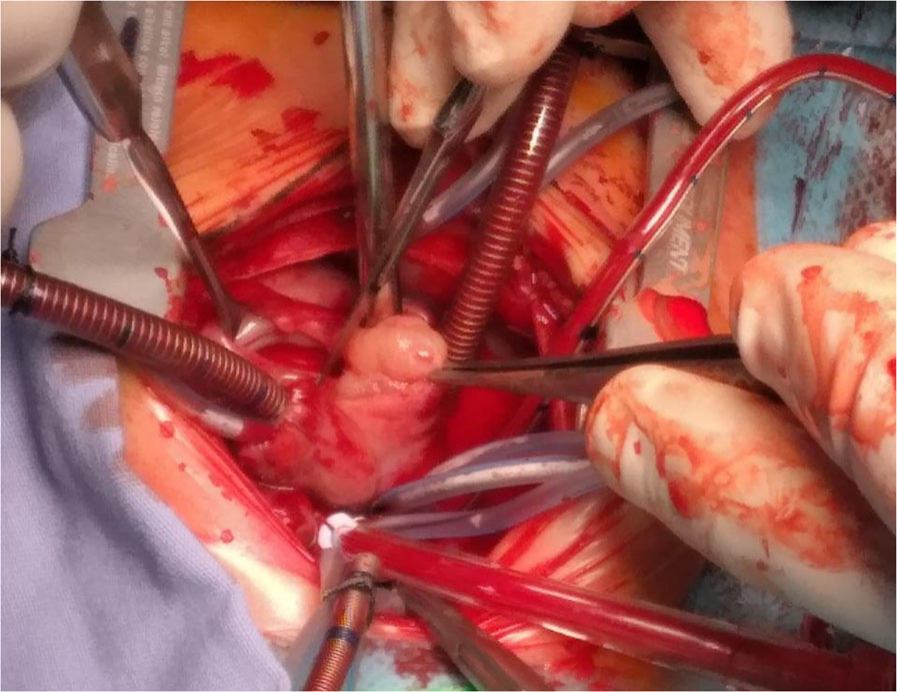
Operative view demonstrates the localization of infiltrative intracardiac Hibernoma

Through median sternotomy and systemic heparinization, the baby was cannulated for cardiopulmonary bypass employing direct bi-caval venous cannulation. After cardioplegic arrest a longitudinal right atriotomy was performed. During surgery, a mass was found to be embedded in the IVS, and to occupy the RV cavity and the right atrium (Fig. 3). The tumor involved also the chordae of the tricuspid. That is why, it was impossible to remove completely the tumor. Partial resection was done. Tricuspid valve repair was performed by construction of new chordae from the autologous pericardium. The baby died immediately after surgery before doing the echocardiography. This evolution is probably due of the right ventricle dysfunction. In fact the tumor was infiltrated the right ventricle and the tricuspid valve. The partial tumor resection of the tumors was insufficient to improve the right ventricle and the restoration of the hemodynamic state. The specimen was sent for histopathological analysis.

**Fig. 4:**
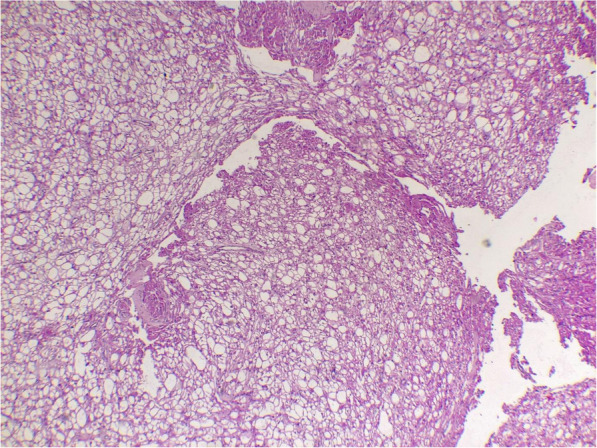
Histologic micrograph shows benign tumor, organized on separated lobule

**Fig. 5:**
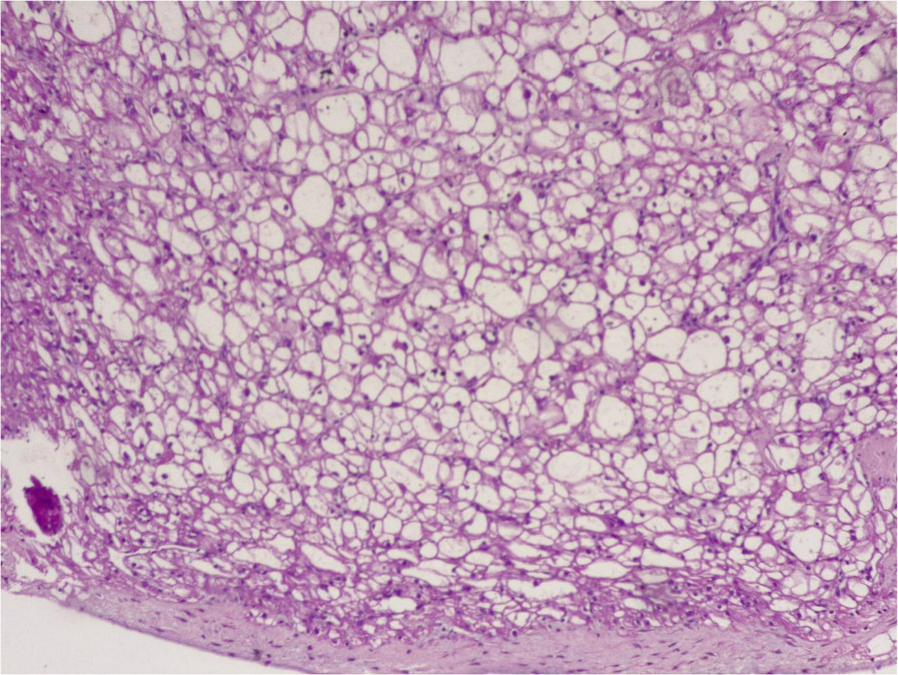
Histologic micrograph shows multivacuolated cells filled with lipid droplets and granular intense eosinophilic cytoplasm

Histological examination of the surgical specimen revealed clear multivacuolated cells filled with lipid droplets and granular intense eosinophilic cytoplasm which confirms the diagnosis of Hibernoma (Figs. 4 and 5). The genetic analysis was not done.

## Discussion

Primary cardiac tumors in newborns are extremely rare and more than 90% of them are benign. They occur approximately in 1 every 100,000 live births [2]. The most frequent tumors of the heart observed in pediatric patients are rhabdomyomas, fibromas and lipomas. Hibernoma is a rare, benign soft tissue tumor of brown fat origin. This is not genetic links. Less than 300 cases have been reported in the literature [3]. Its genesis is thought to be the result of nonspecific trauma to the brown fat cells, such as inflammation or infection. Intracardiac Hibernomas are very rare location and few cases were reported in the literature. Typical location described is the endocardium of the right atrium.

The first case of cardiac Hibernoma reported in the literature was a case of 64-year-old patient with a very rare cardiac Hibernoma located in the right atrium. Transesophageal echocardiography and computed tomography have been shown to be useful for differentiating between benign and malignant tumors. The tumor was excised with the use of cardiopulmonary bypass surgery. Histology confirmed diagnosis of a benign cardiac Hibernoma [4].

Luca Di Tommaso et al. reported a case of 51-year-old woman, with no clinical history, presented for an acute haemoptysis. A computerized tomography (CT) scan revealed a nodule of the lower left pulmonary lobe and a 4-cm lesion involving almost completely the interatrial septum and extending into the right atrium near the superior cava vein. A Magnetic Nuclear Resonance (MNR) excluded any relationship with the superior cava vein. The patient underwent surgery. The definitive histopathology diagnostic was cardiac Hibernoma [5].

Rebeca Mata-Caballero et al. reported a case of 73 -year-old woman, asymptomatic with previous history of breast cancer in complete remission. The tumour was a casual finding on an fluoro-D-glucose integrated with computed tomography (FDG PET/CT). Transthoracic, transesophageal echocardiography showed a mass in the right atrium and aroud the vena cava. Cardiac magnetic resonance help in the differential diagnosis of benign tumor. The mass was excised, allowing the definitive histological diagnosis of benign cardiac Hibernoma. The subsequent outcome was excellent [6].

A case of intrapericardial Hibernoma associated with constrictive pericarditis was also reported. The authors present the case of a 20-year-old male who underwent an operation for the treatment of constrictive pericarditis, in which an intrapericardial sessile lesion over the diaphragmatic surface of pericardial sac was incidentally discovered. The tumor was excised and diagnosed as Hibernoma. No recurrence was evident two years after the procedure [7].

The clinical symptoms vary largely depending on their size, location and mobility of the tumor. It ranges from incidental discovery on imaging tests indicated for other reasons to life-threatening presentations such as cardiac tamponade, arrhythmia, systemic embolization and obstruction. Obstructive symptoms include congestive heart failure, syncope and rarely cardiogenic shock.

To our knowledge, this is the first case report of neonatal cardiac Hibernoma. The fatal issue is due to its large size, the infiltrative and obstruction nature.

In our case the cardiogenic shock was attributed to the obstruction of the right ventricle outflow tract that reduced the cardiac output significantly.

Transthoracic echocardiography (TTE) remains the preferred tool for screening and diagnosing intra cardiac tumors. It allows to specify accurate location, mobility and extent of the tumor. Echocardiography typically reveals a homogeneous, hyperechoic mass, but these findings are not diagnostic of this lipomatous tumor [8].

Magnetic resonance imaging (MRI) and cardiac computerized tomography scan are useful modalities for diagnosis and characterization of intracardiac masses, studying the limits, basis of implantation, and relation ship to surrounding structures.

Cardiac MRI provides the possibility of diagnosing the nature of the tumor by studying its signal features. The specific sign being the complete signal loss of the mass on fat suppression sequence. It is considered to be the diagnostic gold standard [9].

The need for sedation and anesthesia for pediatric population, limits its use in cases of severe symptoms and hemodynamic instability. Such as the case of our patient who was deemed too unstable to undergo sedation and transthoracic echocardiography was sufficient to indicate intervention.

No clear guidelines have been established due to the low incidence of cardiac Hibernoma, but there is consensus that surgical resection of the cardiac tumor is the optimal method of treatment in symptomatic patients. Conservative management may be implemented for asymptomatic patient and prophylactic resection should also be considered [7].

In our case, surgery is indicated and complete resection of the tumor is recommended, but the overgrowth and myocardial infiltration has led to an unsuccessful complete resection.

Long term recurrence of cardiac Hibernoma after surgical resection is possible. Incomplete removal due to diffuse infiltration in the myocardium seems to be the contributing factor for recurrence.

## Conclusion

Cardiac Hibernomas are very rare benign tumors that usually remain asymptomatic. However the prognosis can be unfavorable if the tumor was obstructive and infiltrate the myocardium.

## Data Availability

The data that support the findings of this report are available from cardiology department in Sahloul university Hospital. The author can make it available upon reasonable request.

## Abbreviations

CT: Computerized tomography
FDG: fluoro-D-glucose
LPA: Left pulmonary artery
LV: left ventricle
IVS: Interventricular septum
MNR: Magnetic Nuclear Resonance
MRI: Magnetic resonance imaging
PA: Pulmonary artery
RPA: Right pulmonary artery
RV: Right ventricle
RVOT: Right ventricle outflow tract
TTE: Transthoracic echocardiography
